# Comparison of spatiotemporal and arm swing characteristics of gait between patients with COPD and healthy controls

**DOI:** 10.55730/1300-0144.6112

**Published:** 2025-09-29

**Authors:** Hidaye YAMIKAN, Nihat ÖZGÖREN, Serdar ARITAN, Oğuz KARCIOĞLU, Aynur DEMİREL

**Affiliations:** 1 Department of Cardiopulmonary Rehabilitation, Faculty of Physical Therapy and Rehabilitation, Hacettepe University, Ankara, Turkiye; 2 Department of Exercise and Sport Sciences, Faculty of Sport Sciences, Hacettepe University, Ankara, Turkiye; 3 Department of Internal Medical Sciences, Faculty of Medicine, Hacettepe University, Ankara, Turkiye

**Keywords:** Gait, gait analysis, chronic obstructive pulmonary disease

## Abstract

**Background/aim:**

Impairments in spatiotemporal gait characteristics have been observed in patients with chronic obstructive pulmonary disease (COPD). However, arm swing characteristics—a fundamental component of gait that play a key role in maintaining gait stability and energy efficiency—have been largely overlooked. This study aimed to investigate the spatiotemporal characteristics of gait, together with arm swing patterns, in patients with COPD.

**Materials and methods:**

A total of 20 patients with COPD (aged 40–65 years) and 20 age- and sex-matched healthy controls (HC) participated in this cross-sectional study. Spatiotemporal gait characteristics and arm swing data were recorded using a motion capture system equipped with eight near-infrared cameras during a 6 min walk.

**Results:**

Stride and step length, step time and width, gait speed, and cadence were similar between the groups (p > 0.05). The swing phase ratio was higher in patients with COPD, whereas the gait cycle and stance phase ratios were higher in the HC (p < 0.05). The magnitude and total angular displacement of arm swings were similar between the groups (p > 0.05). In patients with COPD, the right and left arm swings were similar across both the flexion–extension and abduction–adduction axes (p > 0.05), whereas significant right–left differences were observed in the HC group (p < 0.05). Posture quality was significantly poorer in patients with COPD (p < 0.05). The types and presence of scapular dyskinesia were similar between the groups (p > 0.05).

**Conclusion:**

Due to postural impairments and disease-related compensatory adaptations, a more abducted and symmetrical arm swing pattern was observed in patients with COPD. An asymmetrical arm swing pattern was observed in the HC group. These results highlight the importance of assessing and addressing upper extremity movements within rehabilitation programs to optimize gait efficiency and functional mobility.

## 1. Introduction

Chronic obstructive pulmonary disease (COPD) is characterized by systemic inflammation, persistent airflow limitation, and chronic respiratory symptoms, and it is widely recognized as a multisystem disease [1]. It is well established that systemic inflammation affects the respiratory, locomotor, and musculoskeletal systems [2]. Functional and metabolic muscle dysfunctions are observed in both skeletal and respiratory muscles, and ambulation may be adversely affected by reduced oxidative enzyme activity, loss of type I fibers, postural deterioration, and impaired balance [3].

In patients with COPD, alterations in postural alignment and upper extremity positioning are observed, including increased thoracic kyphosis, impaired scapular positioning, and heightened activation of accessory respiratory muscles due to hyperinflation. Hyperinflation may occur even in the early stages of the disease and can increase the work of breathing by altering chest wall compliance. Thoracic and upper extremity kinematics may vary depending on disease severity, degree of airflow limitation, and activation of accessory respiratory muscles [4].

Gait speed is frequently investigated because it is associated with exercise capacity and other clinical outcomes [5]. Gait speed below 0.5 m/s is recognized as a predictive factor for fall risk in patients with COPD [5]. Alterations in spatiotemporal gait characteristics, including reduced cadence, step length, and gait speed, are frequently reported in patients with COPD [6,7].

Gait is a complex, synergistic movement that integrates the coordinated actions of the trunk, lower limbs, and upper limbs [8]. Arm swings contribute to stability, coordination, and gait rhythm in healthy individuals [8]. By reducing vertical angular momentum and energy expenditure, arm swings support the overall stability of human gait [9]. Given the significance of arm swings in gait, their characteristics have been extensively investigated in neurological disorders [10,11]. Although most studies have examined the spatiotemporal characteristics of gait, arm swing dynamics have not been thoroughly investigated in patients with COPD [12,13].

Most studies have focused on gait speed and lower limb kinematics in patients with COPD [14,15]. In particular, gait speed has been frequently investigated because of its feasibility, ease of measurement, and its role as an indicator of independence in activities of daily living [5]. However, gait, as a multidimensional motor activity, involves not only lower limb movements but also upper limb movements [9]. In patients with COPD, upper limb dynamics, which are directly influenced by chest wall mechanics, have been largely overlooked in the literature. Since chest and trunk kinematics are altered by hyperinflation and dyspnea in obstructive diseases such as COPD, upper extremity movements may also be affected. Upper extremity and arm swing kinematics should be investigated since they play a critical role in maintaining gait stability, optimizing energy efficiency, and supporting postural control. Systematic evaluation of arm swing characteristics, integrated with spatiotemporal gait and postural parameters, may contribute to a deeper understanding of the complex nature of gait. From this perspective, the study aimed to compare the spatiotemporal and arm swing characteristics of gait between patients with COPD and healthy controls (HC).

## 2. Materials and methods

### 2.1. Study design

This cross-sectional study was conducted in collaboration between the Hacettepe University, Faculty of Physical Therapy and Rehabilitation, and the Facultyof Sport Sciences, Hacettepe University. The Noninterventional Clinical Studies Ethics Board of Hacettepe University approved this study (approval no: GO 22/903). All evaluations were performed on participants who provided written informed consent prior to participation. This study was conducted in accordance with the Strengthening the Reporting of Observational Studies in Epidemiology (STROBE) guidelines and the principles of the Declaration of Helsinki. The clinical trial number was NCT05746702.

### 2.2. Participants

**Figure 1:**
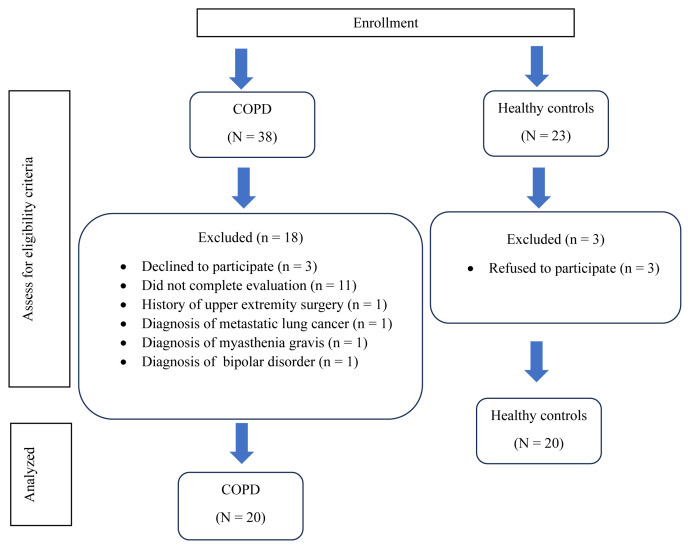
Flow diagram of this study.

Patients diagnosed with COPD according to the Global Initiative for Chronic Obstructive Lung Disease (GOLD) criteria—classified as mild, moderate, or severe—aged 40–65 years, clinically stable for at least the previous 4 weeks, and able to walk independently were included in the study [6,16]. The participant flow chart is presented in Figure 1. Patients with cognitive impairments; those using walking aids or receiving chronic oxygen therapy; and individuals with orthopedic, neuromuscular, neurological, or cardiac conditions affecting posture, upper extremity motion, and arm swing were excluded [6]. Patients with a history of metastasis, thoracic surgery, tuberculosis, sarcoidosis, or asthma were also excluded [6].

### 2.3. Severity of disease

Pulmonary function was assessed using a portable spirometer (Spirodoc; Medical International Research, Rome, Italy) in accordance with the American Thoracic Society and European Respiratory Society guidelines. Disease severity was determined using spirometric measurements and a multidimensional assessment based on the GOLD criteria [17]. According to GOLD criteria, patients were categorized as mild (FEV_1_ ≥ 80% predicted), moderate (50%–80%), severe (30%–50%), and very severe (<30%) based on forced expiratory volume in 1 s (FEV_1_%) predicted. Patients were additionally classified according to the updated GOLD ABE assessment tool into groups A, B, and E based on symptom evaluation, exacerbation risk assessment, and degree of airflow limitation [1].

### 2.4. Posture and posture-related conditions

The same physiotherapist evaluated posture observationally using the Corbin postural assessment scale, rating from 0 (absent), 1 (mild), 2 (moderate), to 3 (severe) based on lateral and posterior views. According to the Corbin posture score, posture is classified as excellent (0–2 points), very good (3–4 points), good (5–7 points), moderate (8–11 points), and poor (≥12 points) [18].

Scapular dyskinesis was assessed observationally by the same physiotherapist and classified according to the Kibler scapular dyskinesis classification. The baseline position was defined as participants standing with their arms relaxed by their sides and their upper bodies unclothed. Participants were instructed to symmetrically elevate both arms to their end range three times, and their scapular movements were recorded. The classification included a prominent inferior medial angle of the scapula (Type I), a prominent entire medial border (Type II), superior elevation of the scapular border (Type III), and symmetrical scapular motion (Type IV) [19].

Scapular position was assessed bilaterally and observationally in three arm test positions: standing neutral, hands on the iliac crest (abduction at 45°), and arm elevation at 90° in the posterior view. The distances between the medial border of the scapula and the T3 spinous process, and between the inferior angle of the scapula and the T7 spinous process, were measured at the end of each of the three positions using a tape measure. A difference greater than 1.5 cm in any of the three positions was accepted as a positive lateral scapular slide test (LSST) result [20].

### 2.5. Gait analysis

**Figure 2a:**
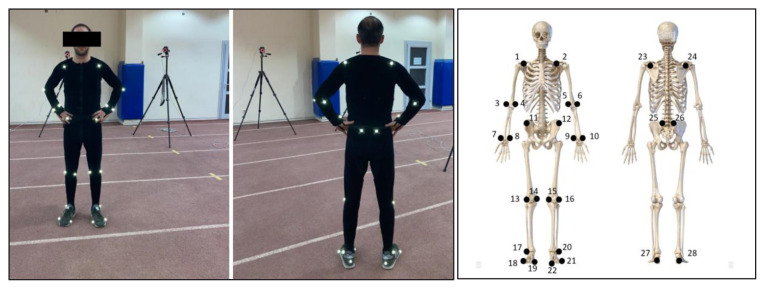
Reflective markers on participants and **Figure 2b**. Twenty–eight reflective markers on anatomical landmarks.

A motion capture system (Vicon Blade, version 2.6; Vicon Motion Systems Ltd, Oxford, UK) equipped with eight near-infrared optical cameras (Bonita model; Vicon Motion Systems Ltd, Oxford, UK) was used to record participants’ gait within a motion capture area measuring 5 m in length and 3 m in width. A total of 28 reflective markers were bilaterally attached to anatomical landmarks on each participant to determine angular kinematics (Figures 2a and 2b) [21,22]. The motion capture area was calibrated using a five-point calibration wand before each recording session. Participants walked at a self-selected comfortable speed for 6 min wearing their own shoes during a single trial. The first and last 5 m of walking were recorded within the motion capture area using Blade software (version 2.61; Vicon Motion Systems Ltd., Oxford, UK) at a sampling frequency of 120 Hz.

Three-dimensional spatial coordinates of the markers were reconstructed and labeled using a custom 14-segment skeleton model created in Blade software. The three-dimensional (3D) coordinates of the markers were exported as TRC files (Vicon track row column format).

**Figure 3a:**
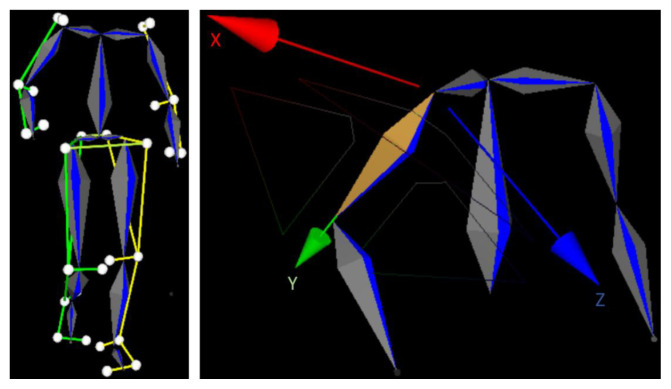
Labelled markers, **Figure 3b**. 14–segment skeleton the local coordinate system of the upper arm segment.

Joint angles were calculated using the built-in inverse kinematics solver in Blade software and exported as BVH files (Biovision Hierarchy format). The raw kinematic data were zero-phase filtered using a second-order low-pass Butterworth digital filter with a 12 Hz cutoff frequency in MATLAB. The arm angle was defined as the angle between the upper arm and the clavicle. The local coordinate system of the upper arm is illustrated in Figures 3a and 3b. Rotations around the X, Y, and Z axes represent flexion–extension, internal–external rotation, and abduction–adduction motions, respectively.

The following spatiotemporal and arm swing parameters of gait were calculated bilaterally: walking speed (m/s), step width (cm), cadence (steps/min), step time (s), stance phase (%), stride length (cm), gait cycle duration (s), step length (cm), swing phase (%), arm swing magnitude (cm), and total arm swing angle (°).

**Figure 4:**
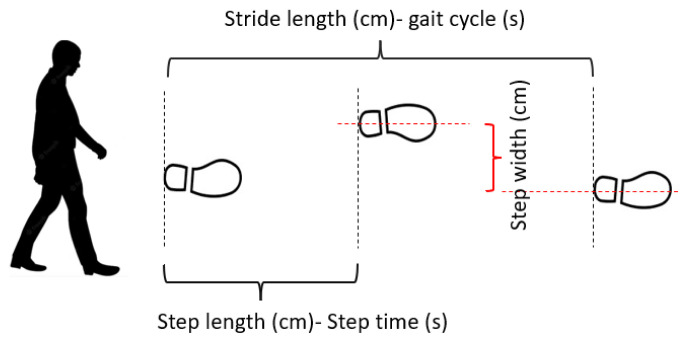
Spatiotemporal gait characteristics.

Stride length, step length, and step width were calculated as distances using heel markers in the anteroposterior and mediolateral directions (Figure 4). Step time, stance phase, swing phase, and gait cycle duration were derived from the 3D position–time trajectories of the heel markers. Swing and stance phases were measured in seconds, and their relative durations were expressed as percentages. Gait speed, cadence, and gait cycle duration were calculated using the following formulas: gait speed = distance (m) / time (s); cadence = (steps × 60) / time (s); and gait cycle duration = (time (s) × 2) / steps [23].

Arm swing magnitude and total arm swing angle were calculated bilaterally within each stride [24]. Arm swing magnitude was defined as the distance between the ipsilateral midpoint of the wrist markers and the midpoint of the pelvic markers in the anteroposterior direction. Accordingly, two peak values of arm swing magnitude were obtained as the arm moved backward and forward.

Arm swing angles around the X and Z axes corresponded to the flexion–extension and abduction–adduction motions of the upper arm, respectively. The cumulative sum of these angular displacements within each stride was calculated and defined as the total arm swing angle in the X and Z axes.

The total arm swing angle around the Y axis represented the rotational motion of the upper arm. As the calculated rotation angles were minimal, they were excluded from statistical analysis.

Gait speed, cadence, stride length, step length, arm swing magnitude, and total arm swing angle were normalized to participant height [25,26].

### 2.6. Statistical analysis

IBM SPSS Statistics software (version 23.0; IBM Corp., Armonk, NY, USA) was used for statistical analyses. Normality was assessed using probability plots, histograms, and the Shapiro–Wilk test. The Student’s t-test was applied for normally distributed data, and the Mann–Whitney U test was used for nonparametric data to determine group differences. The Wilcoxon signed-rank test was performed for within-group comparisons. The descriptive level of significance was set at p < 0.05.

### 2.7. Sample size

Power analysis was conducted using G*Power software (version 3.1; Heinrich Heine University, Düsseldorf, Germany). Sample size estimation was based on preferred walking speed data from Iwakura et al., with α = 0.05, power = 0.80, and effect size = 0.75 [13]. As a minimum of 17 participants per group was estimated; considering potential dropouts, the final sample size was determined as 20 participants per group.

## 3. Results

### 3.1. Participants

A total of 20 patients with COPD and 20 age- and sex-matched HC participated in this study. The COPD group included patients classified according to the GOLD criteria as having mild (50%), moderate (40%), and severe (10%) disease. According to the GOLD ABE assessment tool, 11 patients (55%) were in group A, six (30%) in group B, and three (15%) in group E. According to dyspnea evaluation using the modified Medical Research Council (mMRC) scale, most COPD patients were classified as having mild dyspnea (65%) (Table 1).

### 3.2. Posture and posture-related conditions in patients with COPD and healthy controls

Total posture scores were significantly higher in patients with COPD than in the HC group (p = 0.020). According to postural classification, most patients with COPD had moderate posture (55%), whereas most healthy controls had very good posture (30%). The types and presence of scapular dyskinesis were similar between the groups (p > 0.05) (Table 2).

### 3.3. Spatiotemporal gait characteristics and arm swing patterns in patients with COPD and healthy controls

In patients with COPD, gait cycle duration was shorter than that of the HC group (p = 0.030). The swing phase ratio was higher in patients with COPD, whereas the stance phase ratio was higher in the HC group (p = 0.007). No significant differences were found in stride length, step length, step width, step time, gait speed, or cadence between the groups (p > 0.05). According to between-group comparisons, arm swing magnitude and total arm swing angles in the flexion–extension and abduction–adduction axes were similar between the groups (p > 0.05) (Table 3).

**Figure 5:**
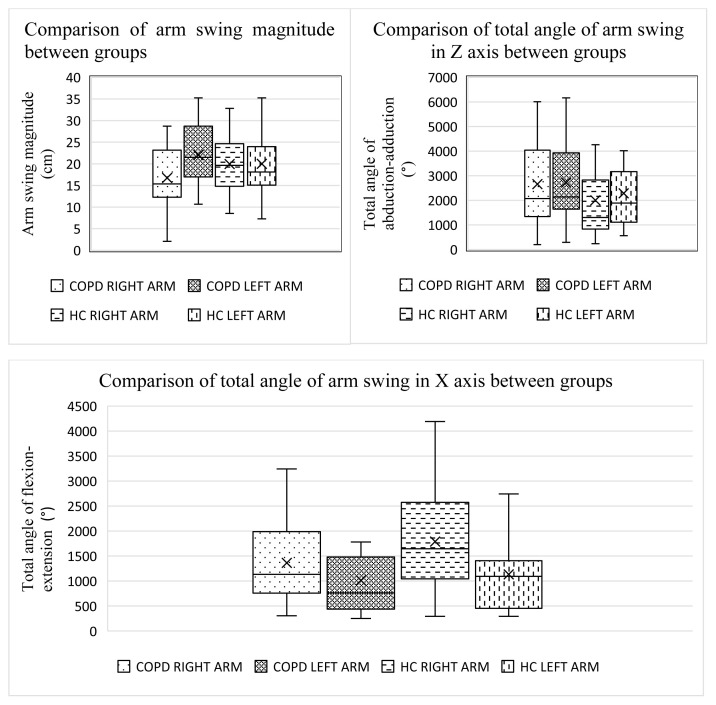
Comparison magnitude and total angle of arm swing between groups.

Within-group analyses showed no significant differences in total arm swing angles between the right and left arms in the flexion–extension or abduction–adduction axes among patients with COPD (p > 0.05). In the HC group, the total angle of the right arm was greater than that of the left arm in the flexion–extension axis (p = 0.004), whereas the total angle of the left arm was greater than that of the right arm in the abduction–adduction axis (p = 0.005) (Table 4). These results indicate that patients with COPD exhibited greater abduction and more symmetrical arm swings during gait. HC exhibited an asymmetric arm swing pattern (Table 4; Figure 5).

## 4. Discussion

The present study compared the spatiotemporal characteristics and arm swing patterns of gait between patients with COPD and HC. The observed tendency toward a more symmetrical and abducted arm swing pattern in patients with COPD, compared with the asymmetrical pattern seen in HC, likely reflects compensatory adaptations to altered thoracic and chest wall mechanics associated with the disease. These findings suggest that gait assessment in COPD should extend beyond conventional measures, such as gait speed and lower limb kinematics, to include systematic evaluation of upper extremity dynamics, as these factors may enhance gait efficiency and reduce the metabolic cost of walking. It is well established that the metabolic cost of walking increases and energy efficiency during gait is impaired in patients with COPD [27,28]. Increased oxygen consumption and perceived exertion during gait negatively affect ambulation and functional capacity in patients with COPD [28]. Altered arm swing kinematics may increase the mechanical work and energetic cost of walking. The present study highlights that optimizing arm swing characteristics may play a critical role in rehabilitation for patients with COPD by reducing the mechanical work of walking, improving gait energy efficiency, and enhancing stability.

The trunk, which coordinates arm and leg movements, is considered the central structure that generates angular momentum during gait [29]. It has been reported that arm swings contribute to gait stability by reducing lateral displacement of the body [30]. When arm swing amplitude was deliberately reduced, the body experienced greater lateral displacement due to increased angular momentum of the swinging leg, thereby impairing gait [30]. In the present study, patients with COPD maintained balance by keeping step width similar to that of the HC group despite reduced arm swing. In addition, a more abducted arm swing pattern in patients with COPD may represent a compensatory strategy to maintain the body’s center of mass. Arm swing movement primarily originates from the trunk rather than the shoulder complex; therefore, a more abducted arm swing pattern is observed. It is suggested that impaired posture may reduce arm swing amplitude in patients with COPD.

Arm and leg movements are regulated by central pattern generators located in the spinal medulla, which are responsible for rhythmic motor activities such as gait [31]. A previous study reported that when leg rhythm changes, arm rhythm adapts accordingly during gait [32]. Gait speed affects the coordination of the legs and arms due to changes in angular momentum [33]. It has been reported that when walking speed increases from 0.75 m/s to 1 m/s, lower extremity momentum rises due to pelvic motion, leading to increased arm swing amplitude [33]. In the present study, patients with COPD may have maintained cadence and step length similar to that of the HC group to balance lower extremity momentum. Therefore, it is possible that patients with COPD developed compensatory adaptations to maintain balance during gait.

Reduced cadence, step length, and self-selected walking speed have been reported in patients with COPD [6,34]. It has been reported that cadence decreases in patients with COPD, allowing individuals with reduced pulmonary function to walk for a longer duration [35]. Conversely, some studies have reported similar spatiotemporal gait parameters in patients with COPD and healthy controls [12,36]. In the present study, cadence was similar between the groups. As most patients with COPD in this study had mild disease severity, a possible explanation may be that lung function is the primary factor influencing cadence.

In normal gait, the stance and swing phases account for approximately 60% and 40% of the gait cycle, respectively [37]. Our findings indicate decreased stance phase and increased swing phase ratios in patients with COPD. As gait speed increases, a shorter stance phase and a longer swing phase are observed in healthy individuals [37]. Patients with COPD demonstrated gait speed and cadence similar to those of healthy controls, which may explain the increased swing phase and decreased stance phase ratios observed in these patients. Patients may develop adaptations specific to the disease. These alterations in the gait cycle may reflect compensatory strategies to maintain gait stability and efficiency. A relatively prolonged swing phase in patients with COPD may indicate difficulties in modulating limb coordination during gait. In contrast, the longer stance phase observed in healthy controls may represent a more efficient postural support mechanism. The reduced stance phase in patients with COPD could increase energy efficiency due to the decreased duration of ground contact. Detailed assessment of phase-related gait alterations may provide new insights into mechanical efficiency and stability in patients with COPD.

Posture is impaired in obstructive diseases such as COPD and cystic fibrosis, even during the early stages of the disease [38,39]. Particularly, the thoracic and cervical regions are affected, with increased thoracic kyphosis, anterior cervical tilt, and scapular protraction and elevation observed in patients with COPD [4]. In this study, patients with COPD had higher posture scores, indicating impaired posture compared with the HC group. The groups were similar and homogeneous regarding the types and presence of scapular dyskinesis. Consistent with previous literature, increased thoracic kyphosis, shoulder protraction, and anterior cervical tilt were observed in patients with COPD. Increased accessory respiratory muscle activity may occur in patients with COPD as a result of thoracic changes and impaired posture [40]. Accessory respiratory muscle activity has been shown to correlate with reductions in FEV_1_/FVC and FEV_1_, indicating disease severity [41].

Scapular motion, which plays a key role in shoulder joint kinematics, deteriorates as a result of alterations in the chest wall [42]. Alterations in the chest wall can affect the acromioclavicular, glenohumeral, and scapulothoracic joints through biomechanical linkage via the sternum [42]. Scapular motion was investigated because it represents a central component of the kinetic chain and may influence arm swing dynamics. No differences were found between the groups regarding the types and presence of scapular dyskinesis; therefore, the homogeneity in scapular dyskinesis allowed for a clearer interpretation of arm swing characteristics.

The principal strength of this study is that it is the first to investigate arm swing and upper extremity movements during gait in patients with COPD. However, several limitations should be considered when interpreting these findings. The main limitation of this study was the potential observational effect within the motion capture area, which may have influenced participants’ natural gait patterns. Participants may have experienced discomfort during gait due to the laboratory environment, the presence of cameras, and reflective markers. Another limitation was that patients with COPD classified as group E declined participation due to exacerbated symptoms during gait and difficulties with mobility and transfer. Therefore, the findings of this study are limited to patients with mild to moderate COPD.

In the present study, an age range of 40–65 years was selected to minimize the confounding effects of age-related changes in gait and arm swing characteristics. Aging has been associated with reduced thoracic and pelvic mobility due to decreased trunk flexibility; therefore, arm swing amplitude tends to diminish [16,43]. By selecting participants aged 40–65 years, we aimed to reduce the influence of extreme age-related biomechanical alterations while encompassing the age range most representative of COPD onset, thereby enhancing the interpretability of gait and arm swing findings in this population.

In conclusion, patients with COPD exhibited more symmetrical and abducted arm swing patterns, whereas healthy controls demonstrated asymmetrical arm swings. Patients with COPD demonstrated a shorter stance phase ratio, a longer swing phase ratio, and a reduced gait cycle duration compared with healthy controls. Posture was impaired in patients with COPD compared with healthy controls. These results demonstrate that arm swing and spatiotemporal gait characteristics are altered in patients with mild to moderate COPD.

Future studies should incorporate detailed assessments of thoracic biomechanics and chest wall kinematics, along with both kinematic and kinetic analyses, to further investigate arm swing mechanisms in patients with COPD. Future research should also include patients with severely impaired airflow (GOLD group E) to evaluate both spatiotemporal gait characteristics and arm swing patterns across the full spectrum of disease severity.

## Figures and Tables

**Table 1:** Participant characteristics.

	COPD (N = 20)	HC (N = 20)	
	X ± SD / Median (IQR)	X ± SD / Median (IQR)	p
Age (years)	59 (50–61)	52 (50–58)	0.207
Sex (F/M)	7 (35)/13 (65)	8 (40)/12 (60)	0.744
BMI (kg/m^2^)	27.16 ± 4.45	27.80 ± 4.32	0.650
Height (cm)	168 ± 10	167 ± 10	0.619
FEV _1_ (% predicted)	78 ± 22/IQR [69–98]	–	–
FVC (% predicted)	90 ± 21/IQR [72–105]	–	–
FEV_1_/FVC (% predicted)	97 (87–101)	–	–
FEV_1_/FVC (L)	75.1 (65.1–77.0)	–	–
GOLD stage (1/2/3) (n/%)	10 (50)/8(40)/2 (10)	–	–
GOLD (A/B/E) (n/%)	11 (55)/6 (30)/3 (15)	–	–
mMRC (Grade 0/1/2) (n/%)	3 (15)/13 (65)/4 (20)	–	–
Walking distance (m)	454.80 ± 64.35	459.30 ± 59.43	0.820

p < 0.05. Data are presented as mean ± SD, median (IQR), or n (%); X: mean; SD: standard deviation; IQR: interquartile range (25–75 percentile); COPD: chronic obstructive pulmonary disease; HC: healthy controls; BMI: body mass index; FEV_1_: forced expiratory volume in 1 s; FVC: forced vital capacity; GOLD: Global Initiative for Chronic Obstructive Lung Disease; mMRC: modified medical research council; CAT: COPD assessment test.

**Table 2:** Posture and posture-related conditions in patients with COPD and healthy controls.

	COPD (N = 20)	HC (N = 20)	
	X ± SD	X ± SD	p
Posture score	8.85 ± 3.76	5.65 ± 4.55	**0.020** *
Posture score classification (n (%))			
Excellent (0–2)	2 (10)	6 (30)	**0.019** *
Very good (3–4)	2 (10)	4 (20)	
Good (5–7)	0 (0)	4 (20)	
Moderate (8–11)	11 (55)	3 (15)	
Poor (12+)	5 (25)	3 (15)	
Scapular dyskinesis types (n (%))			
Type 1	5 (25)	5 (25)	0.962
Type 2	6 (30)	5 (25)	
Type 3	2 (10)	3 (15)	
Type 4	7 (35)	7 (35)	
LSST (n (%))			
LSST 0° (−/ +)	19 (95)/1 (5)	19 (95)/1 (5)	0.998
LSST 45° (−/+)	20 (100)/0 (0)	20 (100)/0 (0)	-
LSST 90° (−/+)	20 (100)/0 (0)	18 (90)/2 (10)	0.147

* Bolded values represent p < 0.05.

Data are presented as mean ± SD or n (%). X: mean; SD: standard deviation; COPD: chronic obstructive pulmonary disease; HC: healthy controls, LSST: lateral scapular slide test.

**Table 3:** Comparison of spatiotemporal gait characteristics and arm swing between patients with COPD and healthy controls.

		COPD (N = 20)	HC (N = 20)	
		X ± SD / Median (IQR)	X ± SD/Median (IQR)	p
Stride length (cm)β	Right	60.06 ± 8.60	59.57 ± 6.34	0.839
	Left	61.12 ± 6.12	58.93 ± 4.89	0.220
Step length (cm)β	Right	21.61 ± 2.54	20.82 ± 3.04	0.380
	Left	22.96 ± 2.77	21.56 ± 2.86	0.124
Step time (s)	Right	0.29 ± 0.06	0.30 ± 0.05	0.640
	Left	0.27 ± 0.06	0.29 ± 0.04	0.332
Step width (cm)		8.86 ± 4.26	9.65 ± 4.00	0.546
Self–selected speed (m/s)β		0.59 ± 0.09	0.57 ± 0.10	0.503
Cadence (steps / min)β		55.35 ± 7.89	50.82 ± 7.18	0.065
Gait cycle duration (s)		1.32 ± 0.15	1.43 ± 0.16	**0.030** *
Stance phase (%)		55.14 ± 3.09	57.87 ± 2.99	**0.007** *
Swing phase (%)		44.85 ± 3.09	42.11 ± 2.99	**0.007** *
Right arm swing (cm)β		9.99 ± 3.91	11.94 ± 3.83	0.120
Left arm swing (cm)β		13.18 ± 4.51	11.97 ± 4.03	0.377
Total right arm–X axis (°)β		677 (439–1107)	961 (628–1565)	0.168
Total left arm–X axis (°)β		436 (270–807)	647 (290–911)	0.482
Total right arm–Z axis (°)β		1288 (838–2099)	792 (541–1582)	0.117
Total left arm–Z axis (°)β		1277 (1011–2356)	1129 (729–1778)	0.358

β Normalized by participant height.

* Bolded values represent p < 0.05.

X: mean; SD: standard deviation; IQR: interquartile range (25–75 percentile); COPD: chronic obstructive pulmonary disease; HC: healthy controls; Total right arm–X axis: total angle of the right arm on the X-axis; Total left arm–X axis: total angle of the left arm on the X-axis; Total right arm–Z axis: total angle of the right arm on the Z-axis; Total left arm–Z axis: total angle of the left arm on the Z-axis; X-axis: flexion–extension motion axis; Z-axis: abduction–adduction motion axis.

**Table 4:** Comparison of total arm swing angle between the right and left arms in patients with COPD and healthy controls.

	COPD (N = 20)	HC (N = 20)
	X ± SD /Median (IQR)	X ± SD/ Median (IQR)
Total right arm–X axis (°)β	677 (439–1107)	961 (628–1565)
Total left arm–X axis (°)β	436 (270–807)	647 (290–911)
p	0.108	**0.004** *
Total right arm–Z axis (°)β	1288 (838–2099)	792 (541–1582)
Total left arm–Z axis (°)β	1277 (1011–2356)	1229 (729–1778)
p	0.550	**0.005** *

β Normalized by participant height.

* Bolded values represent p < 0.05.

X: mean; SD: standard deviation; IQR: interquartile range (25–75 percentile); COPD: chronic obstructive pulmonary disease; HC: healthy controls; Total right arm–X axis: total angle of the right arm on the X-axis; Total left arm–X axis: total angle of the left arm on the X-axis; Total right arm–Z axis: total angle of the right arm on the Z-axis; Total left arm–Z axis: total angle of the left arm on the Z-axis, X-axis: flexion–extension motion axis; Z-axis: abduction–adduction motion axis.
